# Design and Biodistribution of PEGylated Core–Shell X-ray Fluorescent Nanoparticle Contrast Agents

**DOI:** 10.1021/acsami.5c01902

**Published:** 2025-04-23

**Authors:** Giovanni M. Saladino, Bertha Brodin, Mihai Ciobanu, Nuzhet I. Kilic, Muhammet S. Toprak, Hans M. Hertz

**Affiliations:** a Department of Applied Physics, School of Engineering Sciences, KTH Royal Institute of Technology, Stockholm, SE 10691, Sweden; b Department of Radiology, School of Medicine, Stanford University, Stanford, California 94305, United States; c Department of Fiber and Polymer Technology, School of Engineering Sciences in Chemistry, Biotechnology and Health, KTH Royal Institute of Technology, Stockholm, SE 100 44, Sweden

**Keywords:** core−shell nanoparticles, surface functionalization, X-ray fluorescence, PEGylation, nanomedicine, biodistribution, contrast agents

## Abstract

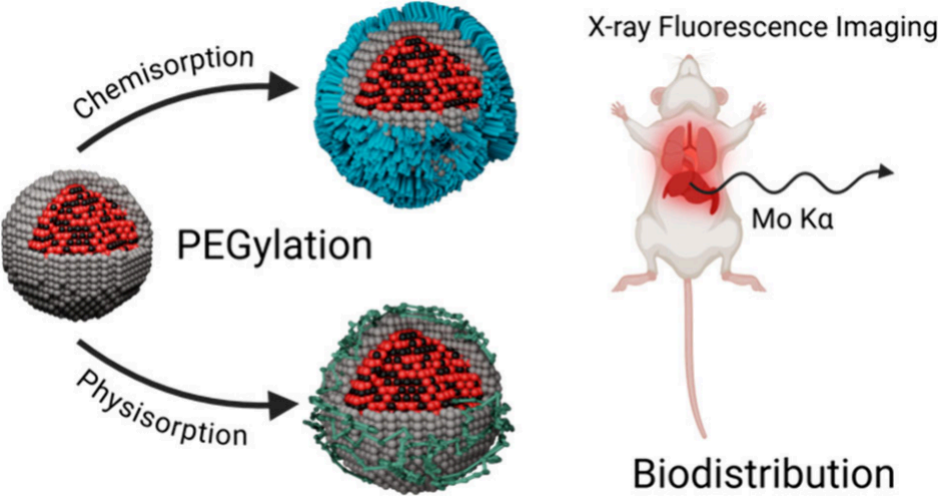

Nanoparticle (NP) uptake by macrophages and their accumulation in undesired organs such as the liver and spleen constitute a major barrier to the effective delivery of NPs to targeted tissues for bioimaging and therapeutics. Surface functionalization with polyethylene glycol (PEG) has been demonstrated to be a promising strategy to limit NP sequestration, although its longitudinal stability under physiological conditions and impact on the NP biodistribution have not been investigated with an *in vivo* quantitative approach. X-ray fluorescence (XRF) imaging has been employed to noninvasively map the *in vivo* biodistribution of purposely designed molybdenum-based contrast agents, leading to submillimeter resolution, elemental specificity, and high penetration depth. In the present work, we design a stepwise layering approach for NP synthesis to investigate the role of chemisorbed and physisorbed PEG on silica-coated molybdenum-based contrast agents in affecting their *in vivo* biodistribution, using whole-body XRF imaging. Comparative quantitative *in vivo* studies indicated that physisorbed PEG (1.5 kDa) did not substantially affect the biodistribution, while the chemisorption route with mPEG-Si (6–9 PEG units) led to significant macroscopic variations in the biodistribution, leading to a reduction in NP uptake by the liver. Furthermore, the results highlighted the major role of the spleen in compensating for the limited sequestration by the liver, microscopically validated with a multiscale imaging approach with fluorophore doping of the silica shell. These findings demonstrated the promising role of XRF imaging for the rapid assessment of surface-functionalized contrast agents with whole-body *in vivo* quantitative pharmacokinetic studies, establishing the groundwork for developing strategies to identify and bypass undesired NP uptake.

## Introduction

Nowadays, the effective delivery of nanoparticles (NPs) to targeted tissues for bioimaging and therapeutics remains a significant challenge.^1^ One of the primary barriers is the uptake of NPs by macrophages, which are key components of the mononuclear phagocyte system (MPS).^2^ Macrophages recognize and clear foreign particles, including NPs, from the bloodstream, significantly reducing their circulation time and bioavailability for intended therapeutic or diagnostic purposes.^3,4^ This process poses a major obstacle to achieving effective targeting in nanomedicine.^5^ The unintended accumulation of NPs in off-target organs, particularly the liver and spleen, further limits their clinical translation.^6,7^ These organs, as primary sites of the MPS, lead to reduced delivery to target tissues and increased risks of systemic toxicity, thus, also raising safety concerns.^8,9^

To overcome these challenges, significant efforts have been directed toward understanding and modulating NP properties to evade immune recognition.^10^ Surface engineering techniques, such as the introduction of polyethylene glycol (PEG) coatings, have been employed to reduce opsonization and macrophage uptake, enhance circulation time, and improve tissue-specific delivery.^11−13^ Nevertheless, the longitudinal stability of PEG coatings under physiological conditions remains poorly understood, due to the limitation of the commonly employed preclinical imaging techniques, including fast radioactive decay, low specificity and limited penetration depth, often requiring *post-mortem* evaluations or precontrast scans.^14−19^ Over time, PEG layers degrade or undergo structural changes, which might be the cause of an increased susceptibility of NPs to immune recognition and inflammatory response.^20−22^

*In vivo* X-ray fluorescence (XRF) imaging is an emerging technique that enables the tracking of NP contrast agents within living organisms.^23,24^ By using a focused X-ray beam from a liquid metal-jet source (24 keV) to excite specific elements (such as molybdenum),^25^ this technique detects the characteristic fluorescence signals emitted, providing spatially resolved maps of elemental distributions *in vivo*, with high sensitivity and submillimeter resolution.^26−28^ We recently employed XRF imaging to study the effect of silica coating on the NP biodistribution using an iterative approach, leading to a reduction of accumulation in the lungs.^29^ In the present work, we propose a stepwise layering approach for the PEGylation of silica-coated molybdenum-based NPs, leading to either physisorbed or chemisorbed PEG on the NP surface. Following the two adsorption mechanisms, we investigated the molecular interactions between PEG and the NP surface, and we established a methodology to quantitatively investigate the NP redistribution upon PEGylation with *in vivo* whole-body XRF imaging. We observed that the chemisorption route resulted in reduced NP uptake in the liver compared to the physisorption route. XRF imaging was proposed as a valuable tool for assessing NP pharmacokinetics, providing whole-body quantitative and longitudinal information on NP biodistribution and accumulation in organs.

## Results and Discussion

### Nanoparticle Design

**Figure 1:**
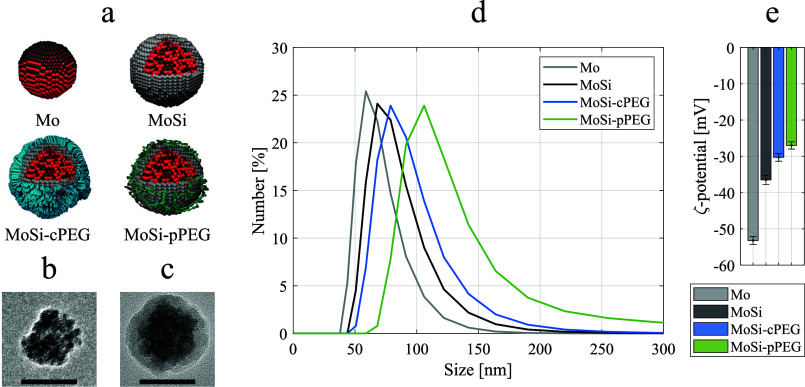
Nanoparticle characterization. (a) Schematic illustration of the multilayer design of the Mo-based contrast agents, leading to molybdenum-based ceramic nanoclusters (Mo), silica-coated nanoparticles (MoSi), followed by surface functionalization with polyethylene glycol through chemisorption (MoSi-cPEG) and physisorption (MoSi-pPEG) approaches. Transmission electron microscopy images of (b) Mo NPs and (c) MoSi NPs. Scale bars, 50 nm. (d) Hydrodynamic size distributions of Mo (in gray), MoSi (in black), MoSi-cPEG (in blue), and MoSi-pPEG (in green). (e) Bar plots of ζ-potential values estimated for Mo (in gray), MoSi (in black), MoSi-cPEG (in blue), and MoSi-pPEG (in green), in triplicates (±SD).

The synthesis of the NPs followed a multilayer design, as schematically shown in Figure 1a and Figure S1. The NP core was constituted of the XRF-active element (molybdenum), obtained through a solvothermal synthesis using ammonium heptamolybdate as the precursor and polyvinylpyrrolidone (PVP) as the capping agent, leading to molybdenum(IV) oxide nanoclusters (Mo NPs).^23^ Their morphology could be visualized through transmission electron microscopy (TEM, Figure 1b) while the thermogravimetric analysis (TGA, Figure S2a) permitted to estimate the inorganic content of Mo NPs as 92.7%. The differential thermogram (DTG) evidenced three peaks, originating from the solvent evaporation (at 105 °C) and two-step PVP pyrolysis (355 and 562 °C).^30^ The elevated inorganic content was beneficial for the maximization of the XRF-active component (Mo) constituting the contrast agent. The XRF spectrum of the Mo NP dispersion was acquired with the *in vivo* imaging setup employing a liquid-metal jet X-ray source at 24 keV (Figure S2b),^25^ highlighting the XRF emission peaks (Mo Kα and Mo Kβ) and Compton scattering (λ_C_), after background removal. The second step in the stepwise layering design was the coating with a uniform inorganic shell of dye-doped (Cy5.5) silica on the nanocluster surface (Figure 1c) leading to MoSi NPs, achieved through a modified Stöber method, using ethanolamine as the base instead of the conventional ammonia, and a purposedly modified Cy5.5 fluorophore.^23^ The silica coating was previously demonstrated to prevent undesired Mo NP accumulations in the lungs, but not sufficient to limit NP sequestration by the liver.^29^ For this reason, the third step was hereby based on the surface functionalization and passivation through PEGylation, pursued through two different approaches: the first conjugation route employed a silane-modified PEG, aiming at achieving chemisorption on the silica surface of MoSi NPs (MoSi-cPEG); the second approach was based on the physisorption of the conventional PEG on the NP surface (MoSi-pPEG). PEGylation through physisorption was achieved by the noncovalent adsorption of PEG, mediated by hydrogen bonding between the silanol groups on the MoSi NP surface and the hydroxyl groups and methylene groups of PEG,^31^ while the chemisorption of mPEG-Si consisted of a covalent condensation of the silane bond on the MoSi NP surface.^32^

All the steps were followed through dynamic light scattering (DLS), which permitted to estimate the hydrodynamic size distribution and polydispersity index (PDI) value after each coating layer (Figure 1d). The bare Mo NPs resulted in an average hydrodynamic size of 67.9 nm (PDI 0.07), while an average size of 81.3 nm (PDI 0.12) was estimated for MoSi NPs, coherently to what was observed in previous studies.^29^ The PEGylation processes led to an increase in the hydrodynamic size, with averages of 91.5 nm (PDI 0.10) and 129.9 nm (PDI 0.19) for MoSi-cPEG and MoSi-pPEG, respectively. The larger overall size of the latter was ascribed to the longer molecular chain of the physisorbed PEG (MW 1.5 kDa) compared to the silane-modified version (6–9 PEG units, 459 – 591 Da). The multilayer design reflected on changes of the surface charge, monitored through ζ-potential (Figure 1e). The PEGylation approaches led to a reduction in absolute values of the ζ-potential, measuring a strongly negative charge for Mo NPs (−53 ± 1 mV) and MoSi NPs (−36 ± 1 mV) and lower absolute values for MoSi-cPEG (−30 ± 1 mV) and MoSi-pPEG (−27 ± 1 mV). This observation was attributed to the partial charge screening of the negatively charged surface of MoSi by the PEGylation. Together with the DLS size distributions, the study of the ζ-potential confirmed the achievement of the surface conjugation with PEG. Phosphate-buffered saline (PBS) is commonly used to assess the hydrodynamic size of NPs intended for *in vivo* applications, as the ionic strength of the biological fluids is a key factor affecting NP stability. The ionic strength of PBS can influence the electrostatic interactions between NPs, thus impacting their aggregation or dispersion behavior in physiological conditions.^33^ We performed a longitudinal study of the hydrodynamic size and PDI of MoSi, MoSi-cPEG, and MoSi-pPEG in PBS. The results showed that all the three NP dispersions were stable within the tested interval (0 – 24 h), with hydrodynamic size values consistently below 150 nm and PDI under 0.3 (Figure S3).

**Figure 2:**
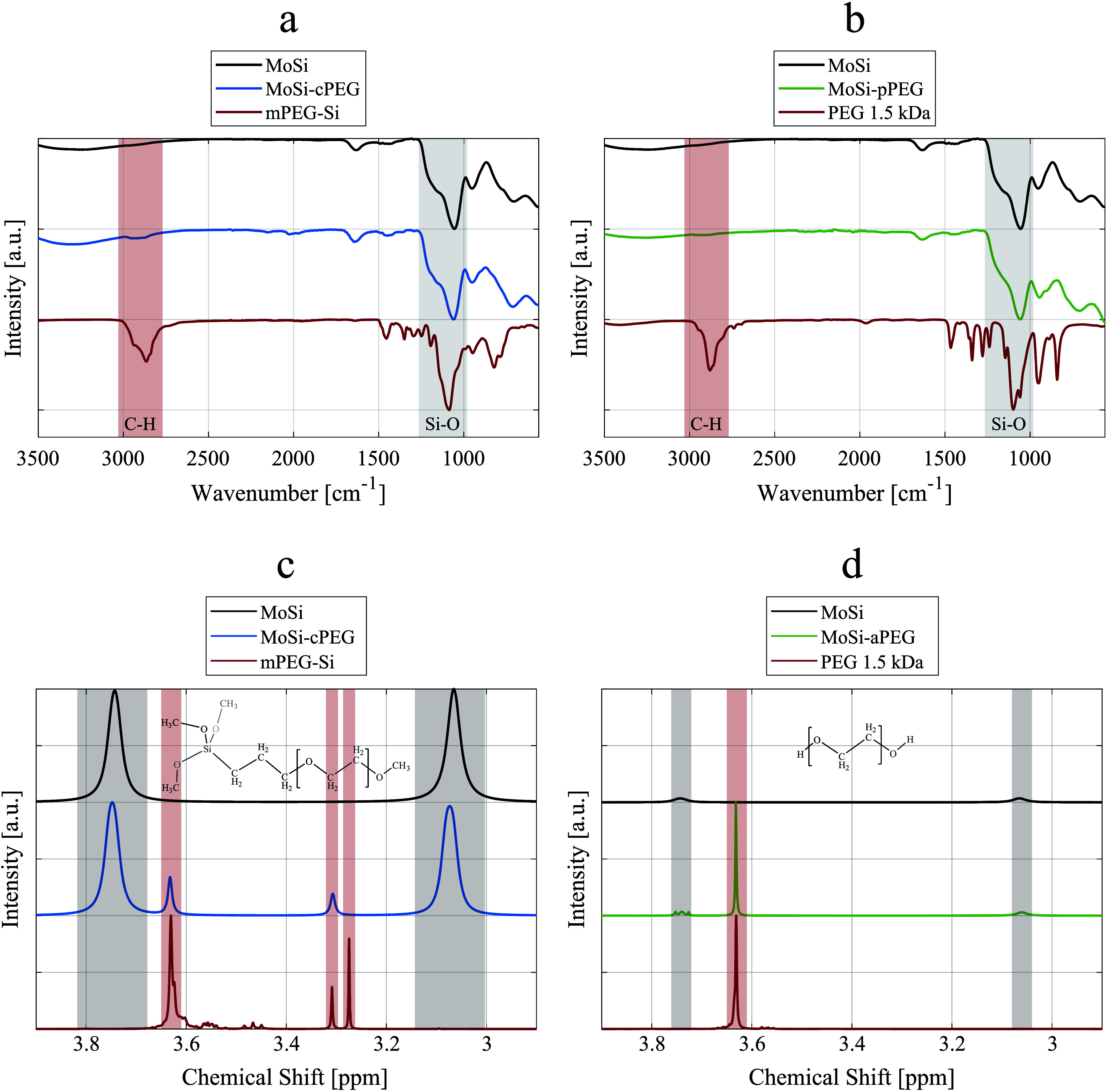
Spectroscopy analysis. FT-IR spectra of (a, b) silica-coated nanoparticles (MoSi, in black), (a) PEGylated nanoparticles through chemisorption (MoSi-cPEG, in blue), (a) 2-[methoxy(6-9polyethyleneoxy)propyl]trimethoxysilane (mPEG-Si, in red), (b) PEGylated nanoparticles through physisorption (MoSi-pPEG, in green), and (b) poly(ethylene glycol) (PEG 1.5 kDa, in red). The most characteristic bands, associated with C–H and Si–O stretching vibrations, were highlighted in red and gray, respectively. Proton (^1^H) NMR spectra of (c, d) silica-coated nanoparticles (MoSi, in black), (c) PEGylated nanoparticles through chemisorption (MoSi-cPEG, in blue), (c) 2-[methoxy(609polyethyleneoxy)propyl]trimethoxysilane (mPEG-Si, in red), (d) PEGylated nanoparticles through physisorption (MoSi-pPEG, in green), and (d) poly(ethylene glycol) (PEG 1.5 kDa, in red). The molecular structures of mPEG-Si and PEG were schematically depicted as the insets.

Scanning electron microscopy (SEM) with energy-dispersive X-ray spectroscopy (EDX) was utilized to confirm the preserved spherical morphology of the PEGylated NPs (Figure S4a). The EDX spectrum exhibited peaks associated with the emission from the main elements constituting the NPs (Figure S4b), O Kα, Si Kα, and Mo Lα, and permitted to estimate the molybdenum/silicon (Mo/Si) elemental ratio, measured as 75:25. Compared to our previous studies, the Mo content was higher owing to the reduced silica precursor (TEOS) amount employed during the silica coating process, coherently with a thinner silica shell.^23^ Fourier-transform infrared (FT-IR) spectroscopy confirmed the presence of the silica shell in MoSi, MoSi-cPEG (Figure 2a), and MoSi-pPEG (Figure 2b) with the specific Si–O stretching vibration band between 980 and 1265 cm^–1^.^34^ Furthermore, the strong bands detected between 2770 and 3030 cm^–1^ in MoSi-cPEG and MoSi-pPEG NPs were associated with the C–H stretching vibrations from the PEG chains.^35^ Finally, ^1^H nuclear magnetic resonance (NMR) spectroscopy was used to investigate whether the two PEGylation methods led to different surface interactions (Figure 2c,d). The NMR spectrum of the free mPEG-Si (Figure 2c), leading to MoSi-cPEG, evidenced three main peaks: the two singlets at 3.27 and 3.31 ppm were assigned to the trimethoxy(methyl)silane group (Si–OCH_3_) and the methoxy terminus of the PEG chain, respectively, while the most intense peak (3.66 ppm) corresponds to the protons of the CH_2_ groups in the PEG chain (−CH_2_CH_2_O−). These assignments align well with the known chemical shifts of methoxy and PEG protons in silane-coupled PEG derivatives.^36^ Coherently, the peak associated with the CH_2_ groups was also found in the free PEG (Figure 2d). The spectrum of the unconjugated MoSi NPs exhibited two broad peaks at 3.15 and 3.74 ppm, attributed to the presence of silanol (Si–OH) groups and residual adsorbed water on the silica surface.^37^

The singlet at 3.66 ppm associated with the PEG chain was preserved in MoSi-pPEG and MoSi-cPEG. For the chemisorption approach, the peak corresponding to Si–OCH_3_ (3.27 ppm) was no longer observed, indicating successful hydrolysis and condensation to form stable Si–O–Si bonds with the NP surface (MoSi-cPEG). The persistence of the peaks at 3.31 and 3.66 ppm further confirmed that the PEG methoxy terminus and backbone remained intact after surface grafting.

Noticeably, the presented chemisorption approach with mPEG-Si was achieved with a one-pot synthesis conjugation method customized based on the employed silica coating method. Using ethanolamine as the base allows to condense silica at lower pH values (<10) than with conventional ammonia (11–13),^23,38^ making the silica coating and PEGylation method more broadly applicable to NPs that exhibit high dissolution and/or oxidation rates in basic media. Overall, the NMR analysis confirmed the successful PEGylation of the NPs. In the future, small-angle X-ray scattering (SAXS) studies could complement the findings made with FT-IR, NMR, and DLS to further investigate the structural differences of the chemisorbed and physisorbed PEG on the NP surface.

### Cell Studies

The synthesized NPs were tested with a cytotoxicity study to evaluate the effect of the multilayer design on the biocompatibility. Real-time cell analysis (RTCA) assay was chosen for it enables continuous, real-time monitoring of cell health and behavior without the need for staining or end point measurements.^39^ RTCA provides a cell index value measured through variations in impedance as a function of time, reflecting cell status without the use of dyes that might interfere with fluorescent NPs.^40^ RAW macrophages were selected as the tested cell line, owing to its fundamental function in NP uptake *in vivo*,^29,41,42^ and its concentration- and time-dependent response to NP exposure.^24^

**Figure 3:**
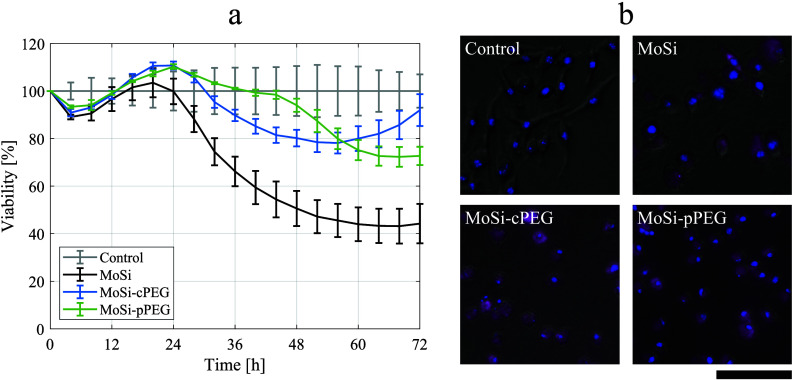
Cytotoxicity study. (a) Real-time cell analysis (RTCA) assay on macrophages (RAW 264.7), after exposure to silica-coated molybdenum- (Mo-) based ceramic nanoclusters (MoSi, in black), MoSi with chemisorbed PEG (MoSi-cPEG, in blue), and MoSi with physisorbed-PEG (MoSi-pPEG, in green), at the same Mo concentration (100 ppm). The viability was obtained from the cell index values normalized to unexposed (negative) control cells (in gray). Measurements were made in triplicates (±SD). (b) Live images of RAW 264.7 macrophages incubated with MoSi, MoSi-cPEG, and MoSi-pPEG ([Mo] = 50 ppm) for 24 h and negative control. Optical fluorescence signal from the nanoparticles (Cy5.5) is shown in pink. Cell nuclei (DAPI staining) are visualized in blue. Trans-luminescence signal is included to visualize cell morphology. Scale bar, 100 μm.

By point-by-point normalizing the cell index values to the ones of unexposed control cells, the RTCA assay permitted to estimate the viability of cells incubated with MoSi, MoSi-cPEG, and MoSi-pPEG NPs (Figure 3a). While no major differences were detected at the earliest times after exposure (*t* < 24 h), a beneficial effect of the PEGylation was observed at longer times, revealing a significant difference compared to the non-PEGylated NPs. MoSi NPs exhibited a half maximal inhibitory concentration (IC50) of 100 ppm (Mo) at 48 h, while the viability of cells exposed to MoSi-cPEG and MoSi-pPEG NPs remained above 70% throughout the study period (72 h) at the tested concentration (100 ppm), with a minor variation between the two PEGylated NP types: after an initial mild cytotoxic effect of MoSi-cPEG, cell viability increased for exposure times exceeding 48 h. This observation might imply that surviving cells proliferated faster than the control, in response to a decreased competition for space, to cover gaps left by detached cells.^43^ This behavior likely indicated a higher cell tolerance to MoSi-cPEG, compared to MoSi-pPEG.

The presented studies highlighted the importance of longitudinal studies when assessing the cytotoxicity of several NP formulations, which were able to reveal delayed cytotoxic responses that would not have been otherwise witnessed. Furthermore, the optical fluorescence property of the Cy5.5-doped silica shell was preserved after PEGylation for both the chemisorption and physisorption approaches, which permitted to track the NPs *in vitro* in exposed macrophage cells with live microscopy images (Figure 3b). The dual NP functionality consisting of XRF and optical fluorescence characteristics enabled by the multilayer design is crucial for a more robust assessment of the NPs with a multiscale imaging approach.^29^

### Biodistribution Studies

**Figure 4:**
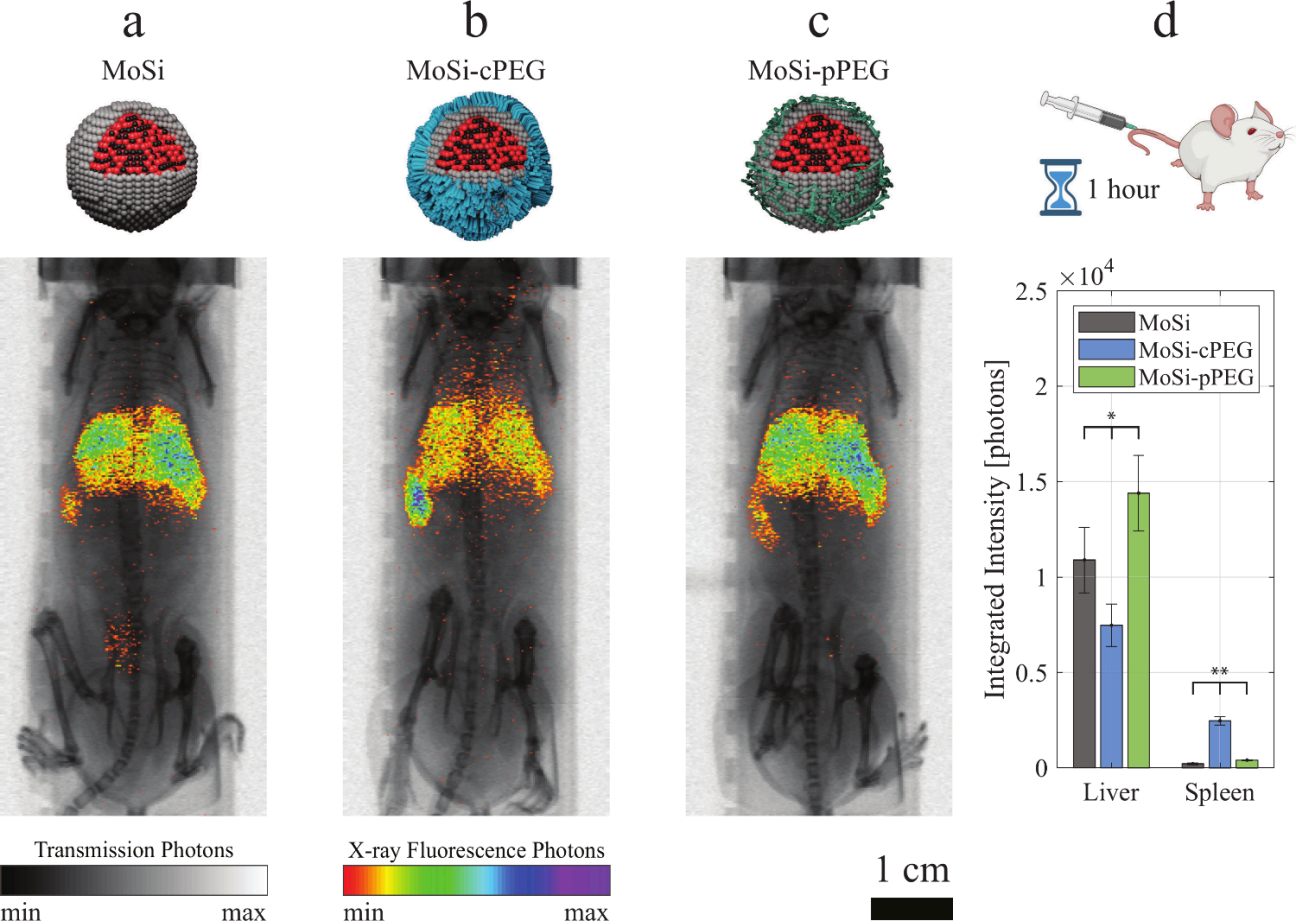
Effect of PEGylation on biodistribution. *In vivo* X-ray fluorescence imaging (XRF) projection images of mice injected with (a) MoSi, (b) MoSi-cPEG, and (c) MoSi-pPEG nanoparticles (NPs). XRF signal (color) was overlaid on X-ray transmission images (grayscale). Images were acquired 1 h after NP injection. Scale bar, 1 cm. (d) Integrated intensity (XRF photons) of 2D-segmented liver and spleen of mice injected with MoSi (in gray), MoSi-cPEG (in blue), and MoSi-pPEG (in green) NPs (*n* = 3, ±SD). Significant difference (ANOVA analysis) between the three groups was indicated when *P < 0.05 and **P < 0.005.

We next investigated the impact of NP PEGylation on the *in vivo* biodistribution using whole-body XRF imaging, chosen for its characteristic elemental specificity (Mo Kα), high sensitivity, and submillimeter resolution.^26^ Uncoated Mo-based nanoclusters were not tested in the present work, as previous studies evidenced undesired accumulations in the lungs.^29^ We earlier demonstrated that our XRF imaging setup provided reliable quantitative estimations of Mo concentrations *post-mortem*, validated with inductively coupled plasma (ICP) mass spectrometry.^44^ In the present work, we proposed an *in vivo* approach to quantitatively assess the effect of PEGylation on the NP biodistribution. The NP biodistribution was studied acquiring XRF projection images at several time points (1 h, 1 day, 1 week) after NP administration (Figures S5–S7). MoSi NPs were employed as the non-PEGylated control, and exhibited major NP accumulations in the liver, detected as XRF photons (Figure 4a, Figure S5). The liver is the primary organ for NP sequestration, due to its extensive blood supply, macrophage armament, and role in filtering and metabolizing foreign entities, with subsequent hepatobiliary clearance.^7,41^ The multilayer design included PEGylation as a known approach to limit NP opsonization and absorption by the reticuloendothelial system.^45^ With the introduced chemisorption route for PEGylation, a macroscopic NP redistribution was observed using *in vivo* XRF imaging (Figure 4b, Figure S6): a reduction in XRF photons from the liver, a higher detection in the spleen, and increased photons in the upper abdomen, likely indicating circulating NPs.

The physisorption approach (MoSi-pPEG) did not lead to a reduction in liver uptake compared to the uncoated MoSi NPs (Figure 4c, Figure S7). A quantitative analysis of the projection images was pursued to identify changes in NP sequestration by the liver and spleen, achieved by the 2D-segmentation of these two organs and subsequent XRF photon integration (Figure 4d). The results evidenced a significant difference in the biodistribution, 1 h after NP injection: the chemisorption route led to a 30% reduction in liver uptake compared to the control, MoSi, which was ascribed to the known role of PEG in limiting opsonization.^11,46^

On the other hand, MoSi-pPEG led to a 30% higher NP sequestration by the liver than MoSi. These findings could be associated with the limited interactions occurring between PEG and the NP surface (Figure 2d), easily perturbed by changes in salinity and pH once the NPs were intravenously administered. Furthermore, the increased overall size (Figure 1d) could have contributed to higher uptake rates, as NPs with larger hydrodynamic diameter are known to have shorter circulation times.^47^

**Figure 5:**
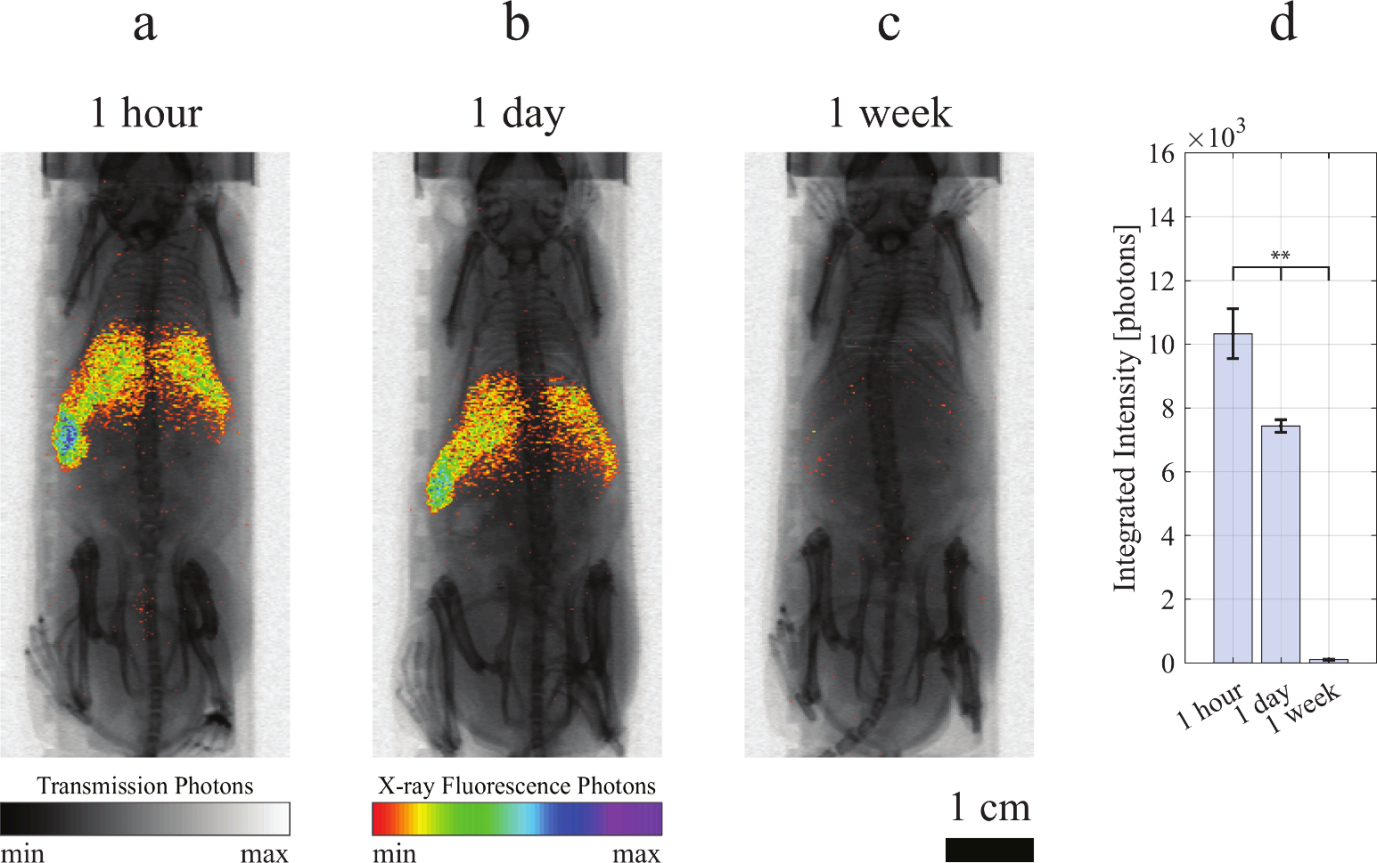
Nanoparticle clearance. *In vivo* X-ray fluorescence imaging (XRF) projection images of mice injected with MoSi-cPEG. XRF signal (color) was overlaid on X-ray transmission images (grayscale). Images were acquired (a) 1 h, (b) 1 day, and (c) 1 week after nanoparticle injection. Scale bar, 1 cm. (d) Integrated overall intensity (XRF photons) of mice injected with MoSi-cPEG (in blue) NPs (*n* = 3:3:2, ±SD), acquired 1 h, 1 day, and 1 week after nanoparticle injection. Significant difference (ANOVA analysis) between the three groups was indicated when **P < 0.005.

One hour after NP administration, the integrated intensity from the spleen regions revealed a significant increment in accumulations of NPs conjugated with the chemisorbed PEG (MoSi-cPEG), registering a 10-fold increase in integrated intensity compared to control (MoSi), while the MoKα signal of mice injected with MoSi-pPEG was only about twice than that of those injected with MoSi NPs. A similar trend was found when performing the analysis on projection images acquired 24 h after the intravenous injection (Figure S8a), evidencing how the biodistribution at early stages was decisive for the NP fate. A longitudinal study with XRF imaging was performed acquiring the projection images at 1 h (Figure 5a, Figures S5–S7), 1 day (Figure 5b, Figures S5–S7), and 1 week (Figure 5c, Figures S5–S7) after injection with the three NP types. The overall integrated intensity (XRF photons) highlighted an almost-complete MoSi-cPEG clearance or residual presence below the sensitivity threshold (Figure 5d), with only 1.0% detected photons at 1 week compared to the first time point (1 h), similarly to MoSi (1.9%, Figure S8b) and MoSi-pPEG (0.6%, Figure S8c) NPs.

This study spotlighted the spleen’s compensatory role in response to partial NP sequestration by the liver. This mechanism underscores the importance of the spleen in the body’s immune and filtration systems, indicating that in cases of targeted NP delivery, splenic involvement must be considered for accurate NP biodistribution and clearance assessments. Several mechanisms are involved in spleen filtration, including mechanical filtration and uptake by marginal-zone macrophages, red pulp macrophages, and sinusoidal endothelial cells.^48^ XRF imaging was demonstrated to be able to quantitatively estimate uptakes in the spleen *in vivo* when comparing several NP types. The proposed stepwise layering design for NP synthesis was able to accurately investigate the effect of each layer on the biodistribution.

### Tissue Immunofluorescence

**Figure 6:**
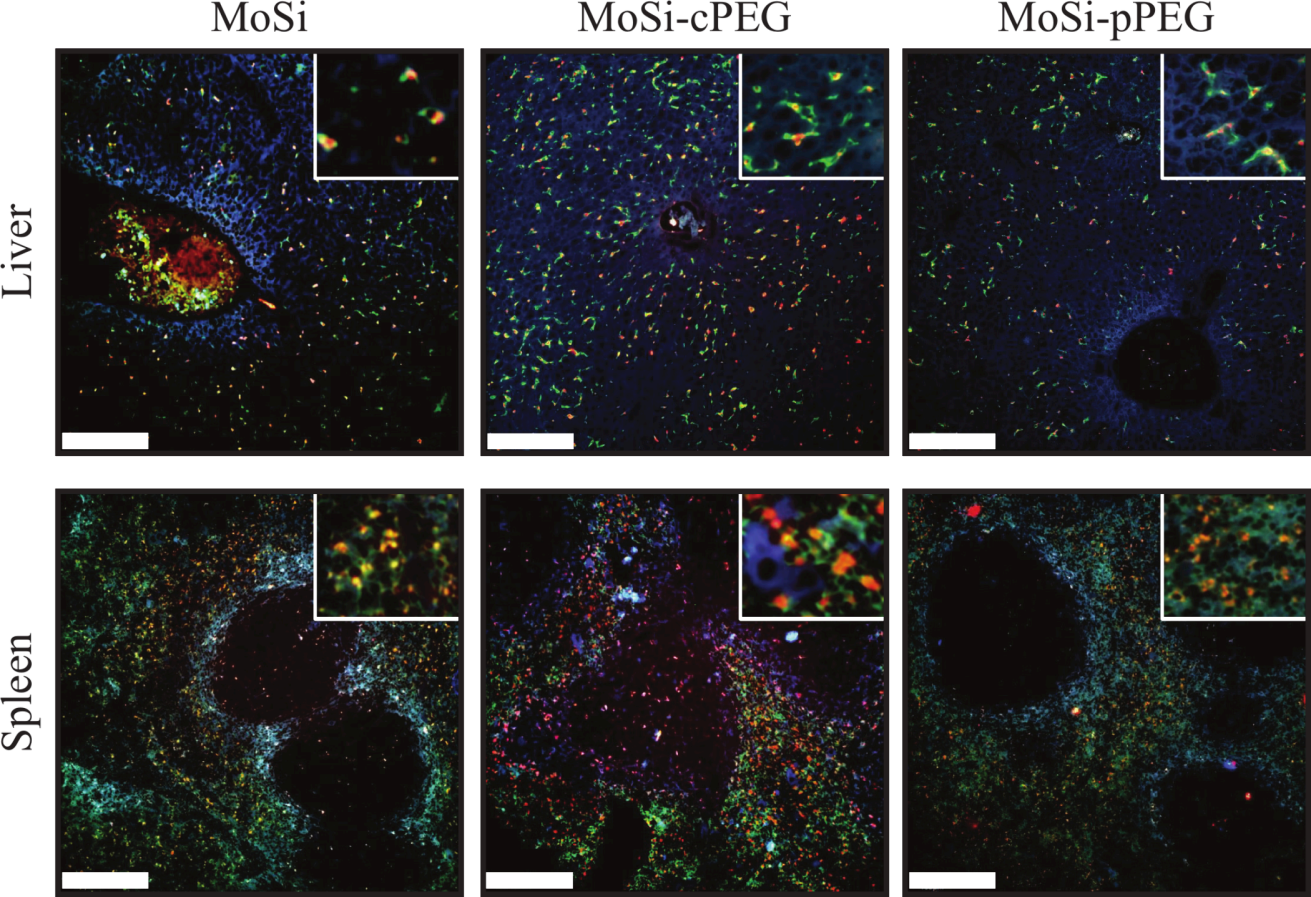
Microscopic analysis. Confocal images of liver (top) and spleen (bottom) tissues of mice intravenously administered with MoSi (left column), MoSi-cPEG (middle column), or MoSi-pPEG (right column) euthanized 24 h after nanoparticle (NP) administration. Immunofluorescence staining (Actin/Phalloidin-Alexa Fluor 405 in blue, F4/80-Alexa Fluor 488 in green, Cy5.5 from NPs in red) at 10× and 63× (insets). Scale bars, 200 μm.

Liver and spleen tissue slides were imaged employing confocal microscopy, after immunofluorescence staining with Actin/Phalloidin and F4/80, to highlight the morphological structure of cells and to identify macrophages, respectively (Figure 6, Figure S9). NP tracking was enabled by the Cy5.5 doping of the silica coating in both MoSi, MoSi-cPEG, and MoSi-pPEG. Cy5.5 was chosen as the dopant because its near-infrared emission reduces background autofluorescence, enhances signal specificity, and provides deeper tissue penetration, making it ideal for NP detection in tissues like the spleen and liver. The tissues were extracted 24 h or 1 week after NP administration and the Cy5.5 signal was mostly colocalized with F4/80 green fluorescence, indicating the major role of liver and splenic macrophages in NP uptake. Limited NP accumulation was detected in the liver and spleen of mice 1 week after administration, indicating that NPs were present below the detection limit of our *in vivo* XRF imaging setup for Mo-based NPs (50 μg/mL).^23^ Interestingly, MoSi-cPEG NPs were also detected in the white pulp of the spleen at 24 h, an area typically low in macrophages. This observation suggested that other cells might have contributed to NP sequestration, especially given the reduced role of Kupffer cells owing to the PEGylation of the MoSi surface. Recent studies revealed that PEGylation of the NP surface may activate other endocytosis mechanisms, clathrin-mediated endocytosis (CME) and caveolin-mediated endocytosis (CAV), besides the most common micropinocytosis.^49^ These alternative uptake pathways might have been the cause leading to a different macroscopic biodistribution of MoSi-cPEG compared to MoSi, tracked through XRF imaging. The presented multiscale approach enabled by the multilayer design and comparative analysis emphasized the importance of rapid whole-body quantitative imaging for the assessment of contrast agents in nanomedicine.

## Conclusions

In the present work, we designed a stepwise layering approach to synthesize NPs as XRF contrast agents and presented a methodology to quantitatively investigate their biodistribution *in vivo*. We studied the effect of each layer on NP pharmacokinetics and highlighted the role of PEGylation in influencing NP uptake by the liver and spleen. Using XRF image analysis, we observed distinct effects between physisorbed and chemisorbed PEG, with the latter leading to a significant reduction in NP sequestration by the liver. Furthermore, a compensatory role of the spleen emerged when the liver exhibited reduced uptake due to PEGylation. In future studies, we will focus on the synthesis of smaller sizes of the core (MoO_2_) and silica shell to enhance circulation times. Furthermore, *in vivo* XRF imaging will provide a reliable tool to investigate the effect of different PEG chain lengths and coatings on enhancing accumulation in specific targets. Overall, these findings underscore the importance of quantitative and comparative assessments in pharmacokinetic studies of nanomedicines, here enabled by whole-body *in vivo* XRF imaging with its high penetration depth, submillimeter resolution, and elemental specificity.

## Experimental Section

### Materials

Ammonium heptamolybdate tetrahydrate (AHM, (NH_4_)_6_Mo_7_O_24_·4H_2_O, analysis grade), poly(vinylpyrrolidone) (PVP, (C_6_H_9_NO)_n_, average MW = 55 kDa), ammonium hydroxide (NH_3_ (aq.) 25%), dimethyl sulfoxide (DMSO, ≥99.9%), triethylamine (Et_3_N, ≥99.5%), tetraethyl orthosilicate (TEOS, >99.0%), (3-aminopropyl) triethoxysilane (APTES, 99%), poly(ethylene glycol) (PEG, average MW = 1.5 kDa), and deuterium oxide (D_2_O, 99.9%) were purchased from Sigma-Aldrich (Sweden). Ethanolamine (EA, >99%), Dulbecco’s Modified Eagle Medium (DMEM), and Iscove’s Modified Dulbecco’s Medium (IMDM) were purchased from Thermo Scientific (Sweden). Ethanol (EtOH, absolute) was purchased from VWR (Sweden). *N*′*N*′-Dimethylformamide (DMF, 99.5%) was purchased from Acros Organics (Sweden). 1*H*-Benz[e]indolium-2-[5-[3-[6-[(2,5-dioxo-1-pyrrolidinyl)oxy]-6-oxohexyl]-1,3-dihydro-1,1-dimethyl-6,8-disulfo-2*H*-benz[e]indol-2-ylidene]-1,3-pentadien-1-yl]-3-ethyl-1,1-dimethyl-6,8-disulfo tripotassium salt (Cy5.5-NHS, 100%) was purchased from Cytiva (Sweden). 2-[Methoxy(6-9polyethyleneoxy)propyl]trimethoxysilane (mPEG-Si, (C_2_H_4_O)_*n*_C_7_H_18_O_4_Si, 90%, 6–9 PEG-units, 459–591 Da) was purchased from abcr GmbH (Germany). All chemicals were used as purchased without further purification. A Milli-Q reference water purification system (Merck Millipore, Burlington, MA, USA) was used for deionized (DI) water.

### Core Nanoparticle Synthesis

The core NPs were synthesized following a previously established method.^23,50^ Briefly, in a Teflon holder AHM (0.356 g) was dissolved in Milli-Q water (56 mL) and EtOH (24 mL). The mixture was stirred for 5 min to dissolve, and PVP (1.189 g) was added. The mixture was stirred for 1 h. The Teflon holder was then enclosed in a stainless-steel casing and placed in an autoclave and heated to 180 °C and for 18 h. After cooling down to room temperature, the mixture was centrifuged and washed with Milli-Q water. The black precipitate was finally redispersed in Milli-Q water (Mo NPs) and stored at 4 °C for further use.

### Silica Shell Coating

The fluorescent dye for silica doping was prepared via a one-pot method, adapted from a previous work.^51^ Briefly, Cy5.5-NHS (1 mg), Et_3_N (0.2 μL), DMSO (50 μL), and APTES (0.3 μL) were added and stirred at room temperature for 24 h. The final product, Cy5.5-APTES, was stored at 4 °C until further use without any further purification. The silica coating procedure is a modified Stöber process previously developed:^29^ to a mixture of EtOH and Milli-Q water (EtOH:H_2_O 3.75:1), Mo NPs (2.85 mg) were introduced and TEOS (10 μL) was added dropwise. The dispersion was stirred for 30 min, then EA (200 μL) was added dropwise to initiate the reaction, immediately followed by the addition of Cy5.5-APTES (1 μL). The mixture was stirred for 2 h at room temperature in a dark environment. The product was centrifuged and washed with EtOH and Milli-Q water. The final precipitate, MoSi NPs, was either dispersed in Milli-Q water or used for the following steps.

### PEGylation

The chemisorption approach was designed following a similar approach as the silica coating. In a three-neck flask, EtOH and Milli-Q water (EtOH:H_2_O 3.75:1) were added and stirred. To the mixture, all the washed MoSi NPs from the previous step were dispersed, and mPEG-Si (1.3 mL) was subsequently added. The solution was stirred for 30 min, followed by the addition of EA (200 μL), acting as a catalytic promoter for the reaction. The mixture was stirred for 2 h at room temperature in a dark environment. The product was centrifuged with subsequent cycles of EtOH and Milli-Q water. The final precipitate, MoSi-cPEG NPs, was dispersed in Milli-Q water and stored at 4 °C for further use.

For the physisorption approach, PEG 1.5 kDa (620 mg) was dispersed in Milli-Q water (10 mL), followed by the addition of the washed as-made MoSi NP. The dispersion was stirred at room temperature for 24 h and the final product was centrifuged with EtOH and Milli-Q water. The final precipitate, MoSi-pPEG NPs, was dispersed in Milli-Q water and stored at 4 °C for further use.

### Characterization Techniques

The dry particle size and morphology of the designed NPs were studied using transmission electron microscopy (TEM, JEM-2100F, 200 kV, JEOL). The hydrodynamic size of the NPs was investigated using dynamic light scattering (DLS, Zetasizer Nano ZS90, Malvern Panalytical), dispersed and diluted in their respective solvents in polystyrene (PS) cuvettes, and the results were reported based on the number-average and PDI values. The inorganic content of Mo-based NPs was estimated via thermogravimetric analysis (TGA, TGA550, TA Instruments), after vacuum-drying the NP dispersion at 40 °C for 48 h. The surface charge of the NPs was studied by ζ-potential (Zetasizer Nano ZS90, Malvern Panalytical) measurements of samples dispersed in Milli-Q water. The NP concentration (Mo) was estimated through XRF measurements with Mo standard dilutions and background removal.^52^ For NP surface characterization, Fourier transform–infrared (FT-IR, Thermo Scientific Nicolet iS10) and ^1^H nuclear magnetic resonance (^1^H NMR, Bruker Avance 400 MHz) spectroscopies were employed. FT-IR spectra were acquired using the attenuated total reflectance (ATR) measurement technique after drying the NP dispersions on a glass slide and inverting the slide to perform the measurements. Ambient atmosphere was used as the background in the spectral range of 600–4000 cm^–1^. For ^1^H NMR, each sample (10 mg) was dispersed or dissolved in D_2_O (0.5 mL), and the spectra were acquired as averages of 14 scans with 2 dummy scans, a 30 s relaxation delay, and a spectral width of 12 ppm.

### Cell Studies

The real-time cell analysis assay (xCELLigence Agilent, St Clara, USA) was employed to investigate the role of physisorbed or chemisorbed PEG on the NP surface. The assay was performed on RAW 264.7 (ATCC-TIB-71) cell line exposed to MoSi, MoSi-cPEG, or MoSi-pPEG, keeping the same Mo concentration (100 ppm) in triplicates (96-well plate, biological replicates, ± SD). Untreated cells were the negative control. The estimated viability was based on the quantification of the impedance, an indicator of cell proliferation, and normalization to the control cell values. The cells were allowed to adhere to the plate surface for 24 h before exposure to the compound (time = 0). Live images of the cells were obtained using an EVOS 5000 Imaging System (Thermo Fisher Scientific, MA, USA), after DAPI staining.

### Animal Studies

Experiments with mice were approved by the regional animal ethics committee of Northern Stockholm, Sweden (ethical permit number 13156-2022), according to institutional, national, and European guidelines for animal handling and research (L150/SJVFS 2019:9 and 2010/63/EU). Eight-week-old female albino mice (BALB/c) were obtained from Janvier Laboratories (France) and housed under controlled temperature (21 ± 1 °C) and humidity (55 ± 5%) conditions, with light–dark cycle and *ad libitum* feeding. The general conditions of the mice were assessed before and during the study, checking for possible onsets of behavioral and/or morphological changes. The mice (n = 3, per group) were intravenously injected with MoSi, MoSi-cPEG, or MoSi-pPEG NP dispersions (20 mg/kg, 100 μL PBS).

### X-ray Fluorescence Imaging

Whole-body XRF projection images were acquired *in vivo* with our laboratory liquid-metal jet X-ray source for XRF imaging.^29^ XRF scans were performed under anesthesia with isoflurane (Abbott, Sweden) at several time points (1 h, 1 day, and 1 week). During the imaging sessions, ophthalmic ointment (Oculentum simplex, APL, Sweden) was applied to the eyes for cornea protection; temperature and respiration were also monitored. A step size of 200 μm and exposure time of 10 ms per step were chosen, resulting in a scanning time of 15 min. For the quantitative analysis, the liver and spleen regions in the whole-body projection image were manually segmented and the XRF photons were integrated within these regions. The analysis of variance (ANOVA) test was employed as the statistical test to analyze the difference between the three groups. Significant difference was indicated when **P < 0.005 and *P < 0.05.

### Histological Analysis

Mice were euthanized by carbon dioxide (CO_2_) inhalation, 24 h or 1 week after NP administration. Liver and spleen were excised and fixed in 4% buffered paraformaldehyde (PFA) solution. After 24 h in PFA solution, organs were transferred into 70% ethanol for storing. By using a rotary microtome, formaldehyde fixed paraffin embedded (FFPE) 4-μm-thick organ sections were obtained and mounted on standard object glasses. After deparaffinization and rehydration, immunofluorescence was performed on the sections using an F4/80 antibody (Alexa Fluor 488) and Actin/Phalloidin (Alexa Fluor 405). Images were obtained using a CrEST X-Light V3 spinning disk microscope (Nikon Instruments Inc., USA) with three active laser lines at 405, 477, and 637 nm and emission filters. Two objectives were used for image acquisition (10× air, 60× oil).

## Supporting Information Available

Nanoparticle characterization, projection images, and additional quantitative analyses (PDF)
