# Supplementary material for: Design and Biodistribution of PEGylated Core–Shell X-ray Fluorescent Nanoparticle Contrast Agents
Source: ACS Appl Mater Interfaces. 2025 Apr 23;17(18):26338–47. doi: 10.1021/acsami.5c01902 (PMC12067382; doi:10.1021/acsami.5c01902)
Supplement: Supplementary file 1 — am5c01902_si_001.pdf [file am5c01902_si_001.pdf]

Supporting Information

Design and Biodistribution of PEGylated Core-Shell X-ray Fluorescent Nanoparticle Contrast Agents

Giovanni M. Saladino,[ab]* Bertha Brodin,[a] Mihai Ciobanu,[a] Nuzhet I. Kilic,[c] Muhammet S. Toprak,[a] Hans M. Hertz[a]

[a] Department of Applied Physics, School of Engineering Sciences, KTH Royal Institute of Technology, Stockholm, SE 10691, Sweden
[b] Department of Radiology, School of Medicine, Stanford University, Stanford, CA 94305, USA
[c] Department of Fiber and Polymer Technology, School of Engineering Sciences in Chemistry, Biotechnology and Health, KTH Royal Institute of Technology, Stockholm, SE 100 44, Sweden

*Corresponding author. Email: saladino@kth.se

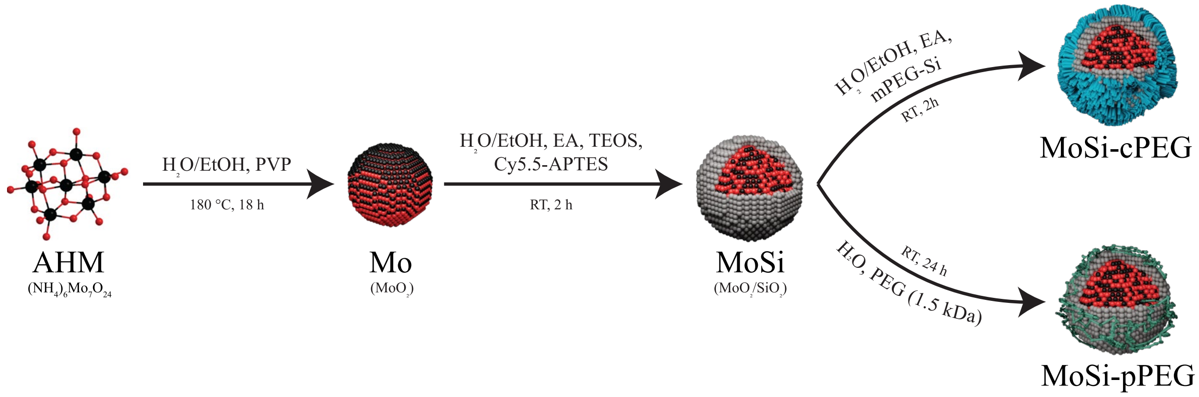

Fig. S1.

Nanoparticle Synthesis Scheme. Mo NPs (MoO_2) capped with polyvinylpyrrolidone (PVP) are synthesized with a solvothermal method using AHM as the precursor. MoSi are obtained by coating Mo NPs with a dye-doped silica (SiO_2) shell, which is condensed on Mo NPs with a modified Stöber method, using TEOS, Cy5.5-APTES, and EA as the silica precursor, dopant, and base, respectively. MoSi NPs were chemisorbed with PEG (MoSi-cPEG) by reaction with mPEG-Si in water/ethanol (H_2O/EtOH) mix. Physisorption of PEG was instead achieved by incubating MoSi NPs with PEG (1.5 kDa) in water for 24 h.

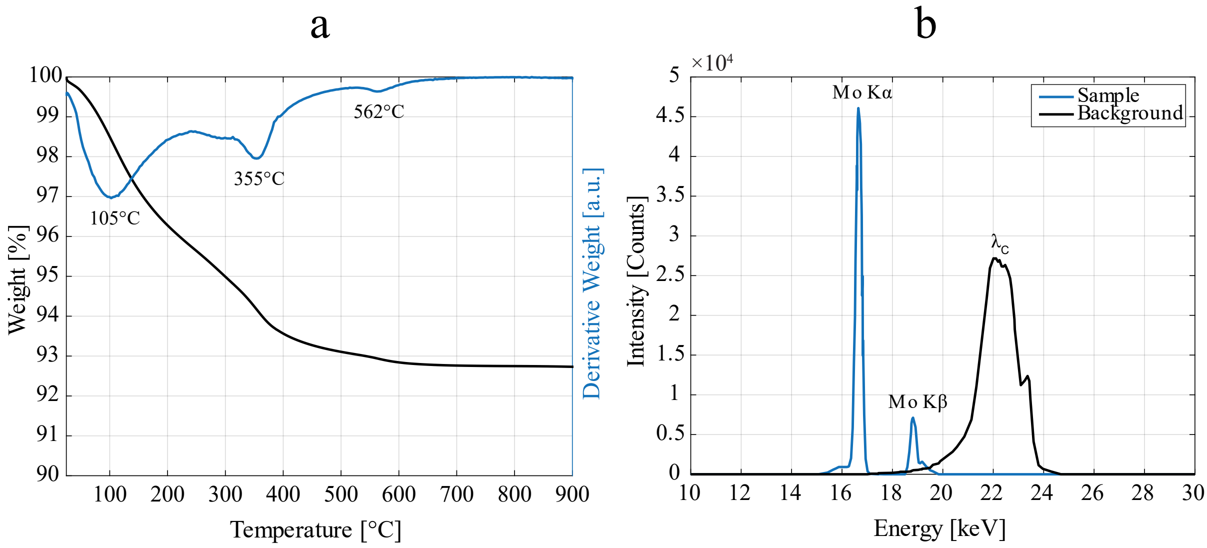

Fig. S2.

Core Nanoparticle Characterization. (**a**) Thermogravimetric (TGA, in black) analysis of molybdenum-based core nanoparticles (Mo NPs) and differential thermogram (DTG, in blue). (**b**) X-ray fluorescence (XRF) spectrum of a Mo NP sample (in blue), after background removal (in black). XRF emission peaks (Mo Kα, Mo Kβ) and Compton scattering (λ_C) are highlighted.

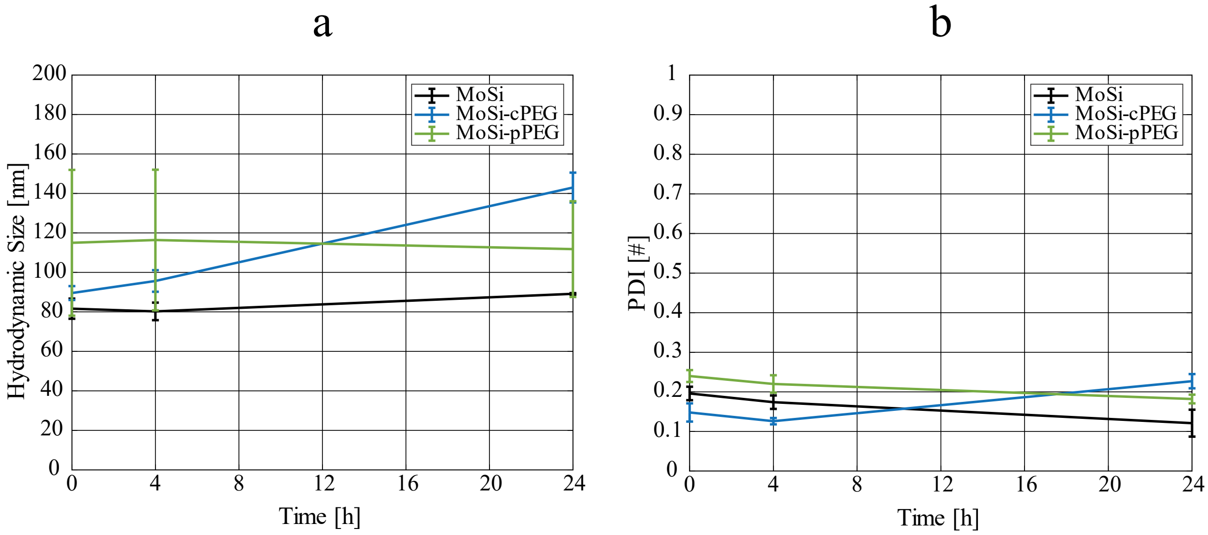

Fig. S3.

Nanoparticle Stability Study. Time-dependent (**a**) hydrodynamic size and (**b**) polydispersity index (PDI) of MoSi (in black), MoSi-cPEG (in blue), and MoSi-pPEG (in green), dispersed in PBS (pH 7.4).

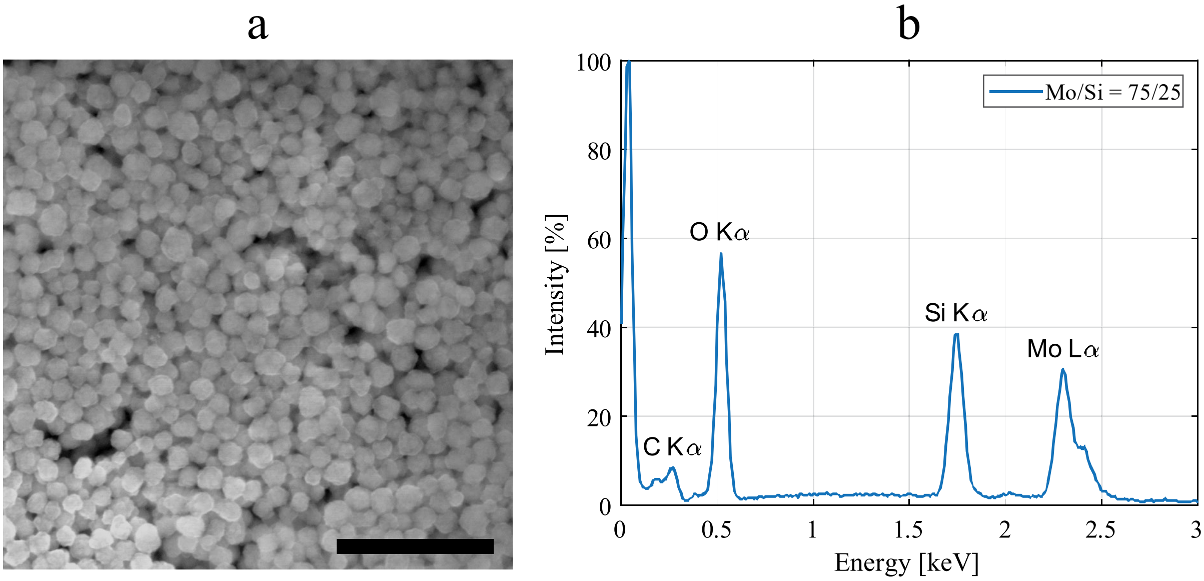

Fig. S4.

Morphological and Elemental Analysis. (**a**) Scanning electron microscopy (SEM) image of PEGylated silica-coated Mo-based nanoparticles (MoSi-cPEG). Scale bar, 400 nm. (**b**) Energy-dispersive X-ray (EDX) spectra acquired with SEM, indicating the major peaks and quantitative elemental ratio between molybdenum and silicon (Mo/Si).

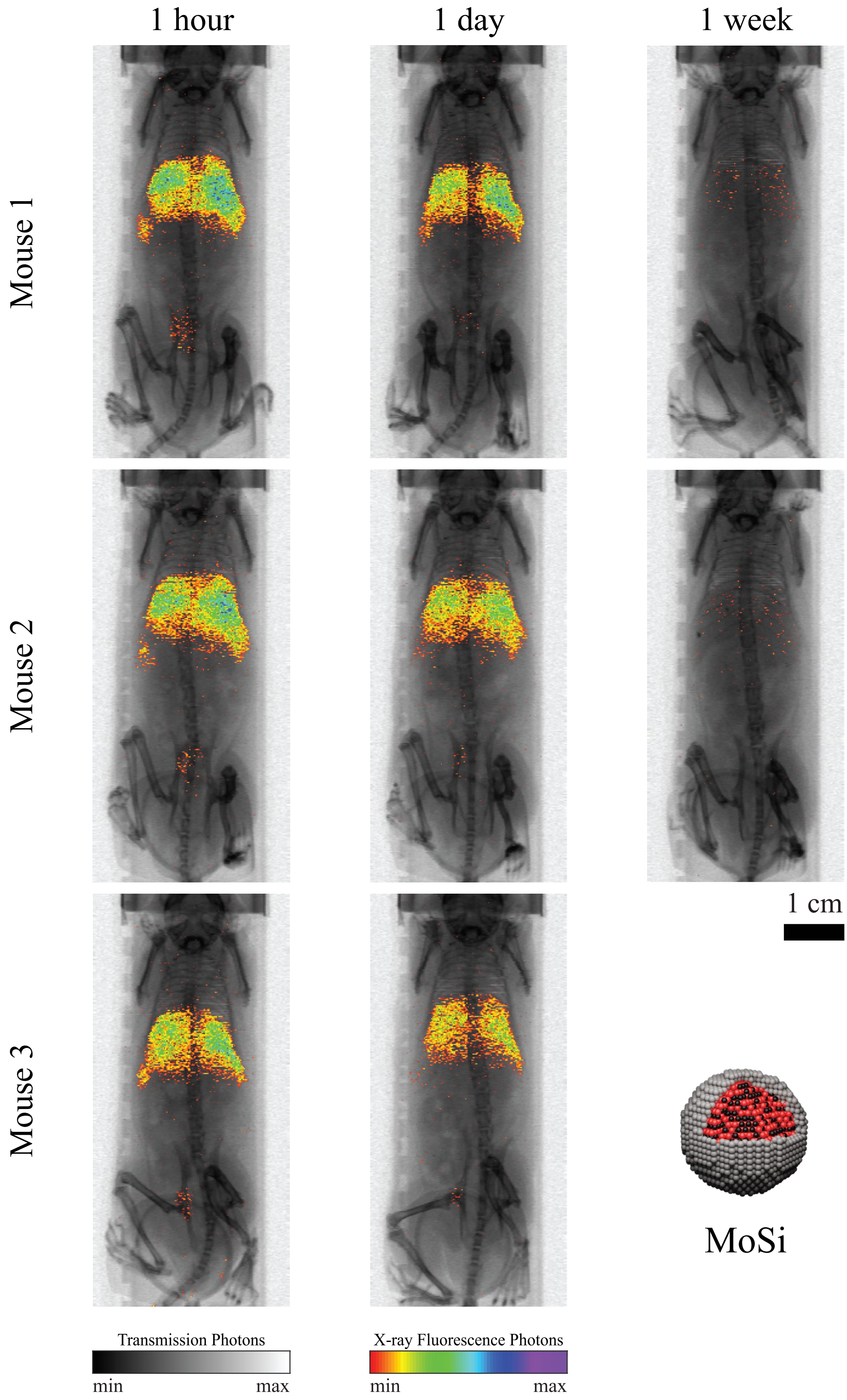

Fig. S5.

X-ray fluorescence (XRF) imaging with MoSi. *In vivo* XRF projection images of mice injected with silica-coated molybdenum-based nanoparticles (MoSi), acquired 1 h, 1 day, and 1 week after NP intravenous administration ([Mo] = 20 mg/kg). Scale bar, 1 cm. XRF signal (color-scale) overlaid on transmitted photons (greyscale).

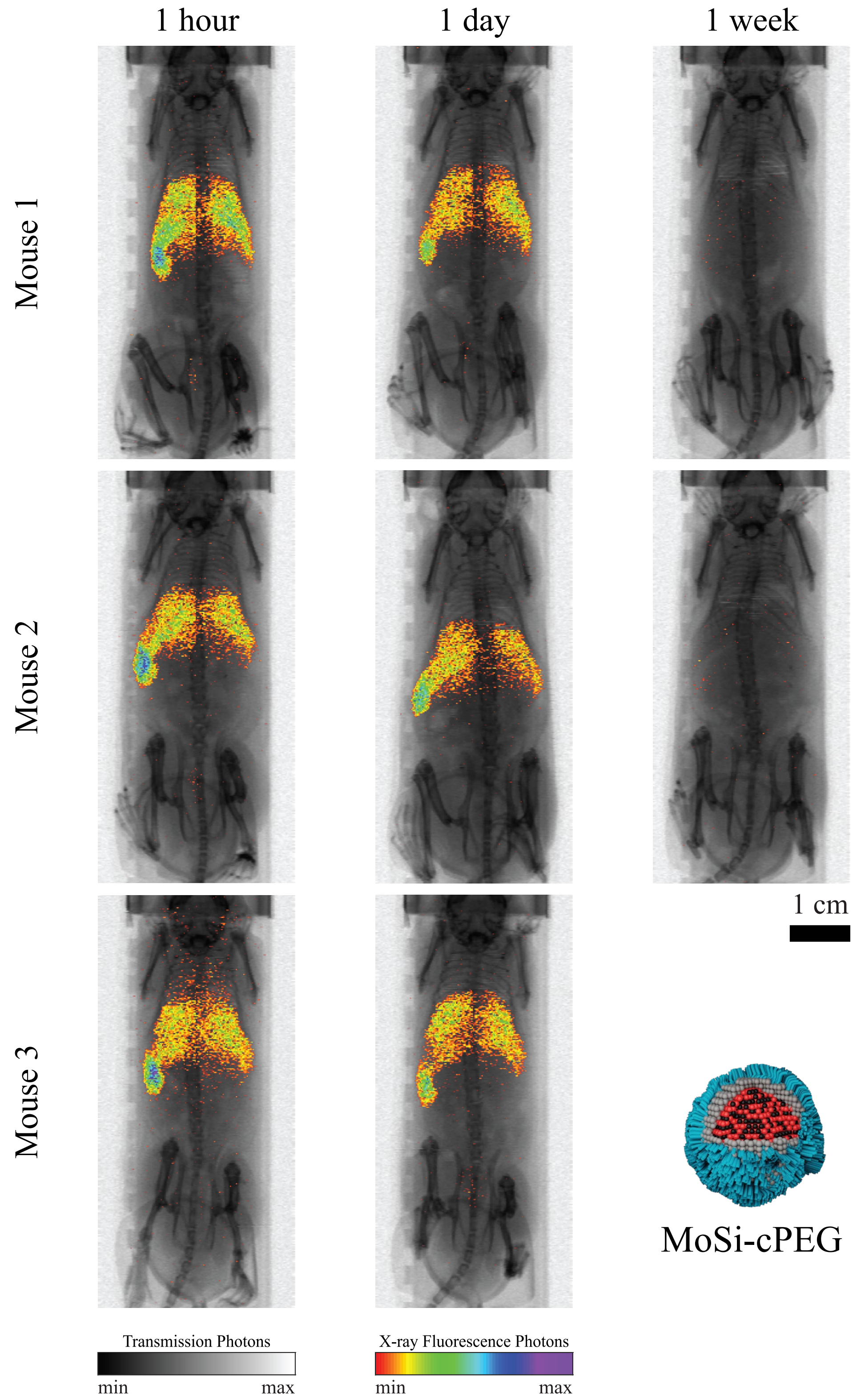

Fig. S6.

X-ray fluorescence (XRF) imaging with MoSi-cPEG. *In vivo* XRF projection images of mice injected with silica-coated molybdenum-based nanoparticles PEGylated through chemisorption (MoSi-cPEG), acquired 1 h, 1 day, and 1 week after NP intravenous administration ([Mo] = 20 mg/kg). Scale bar, 1 cm. XRF signal (color-scale) overlaid on transmitted photons (greyscale).

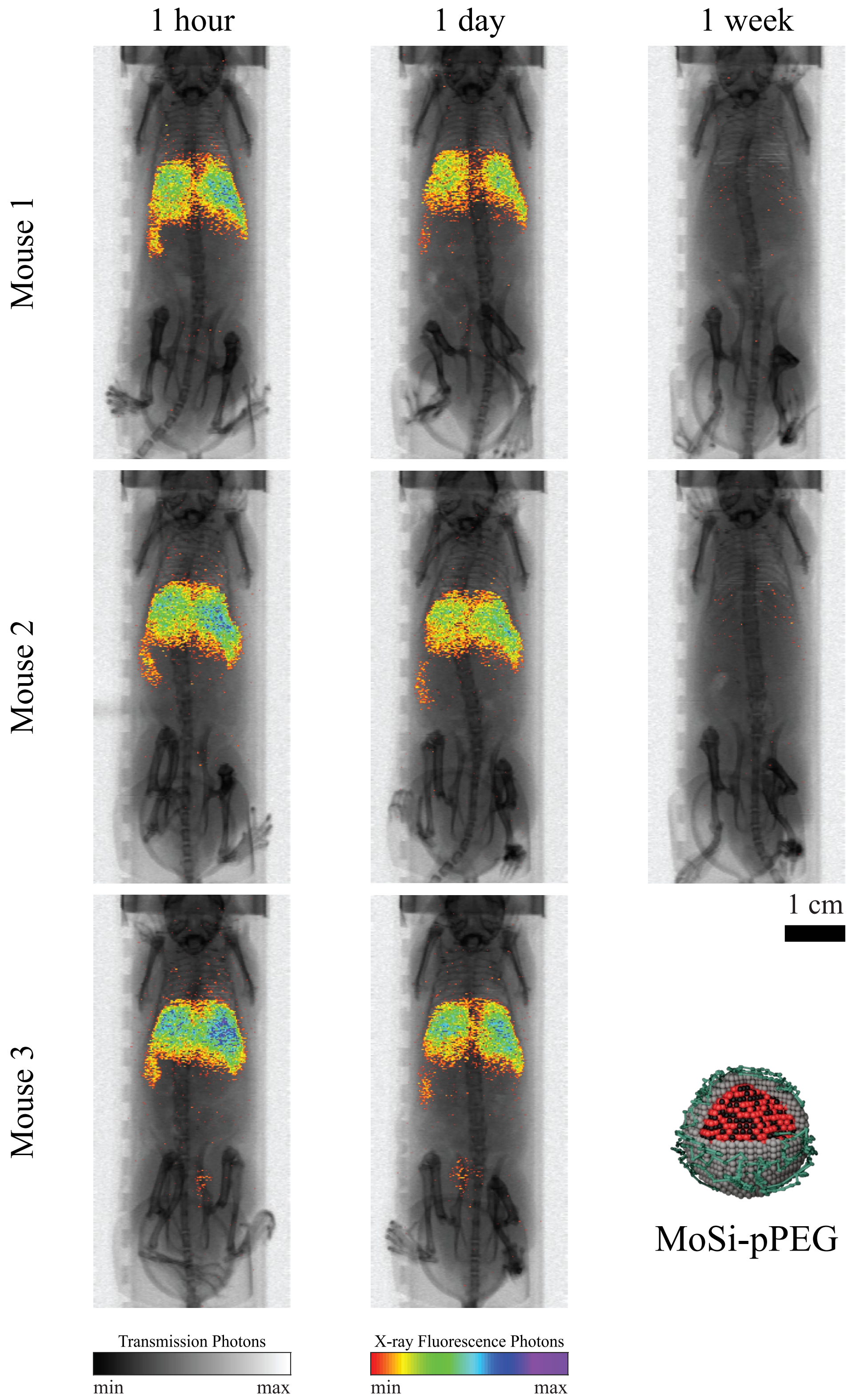

Fig. S7.

X-ray fluorescence (XRF) imaging with MoSi-pPEG. *In vivo* XRF projection images of mice injected with silica-coated molybdenum-based nanoparticles PEGylated through physisorption (MoSi-pPEG), acquired 1 h, 1 day, and 1 week after NP intravenous administration ([Mo] = 20 mg/kg). Scale bar, 1 cm. XRF signal (color-scale) overlaid on transmitted photons (greyscale).

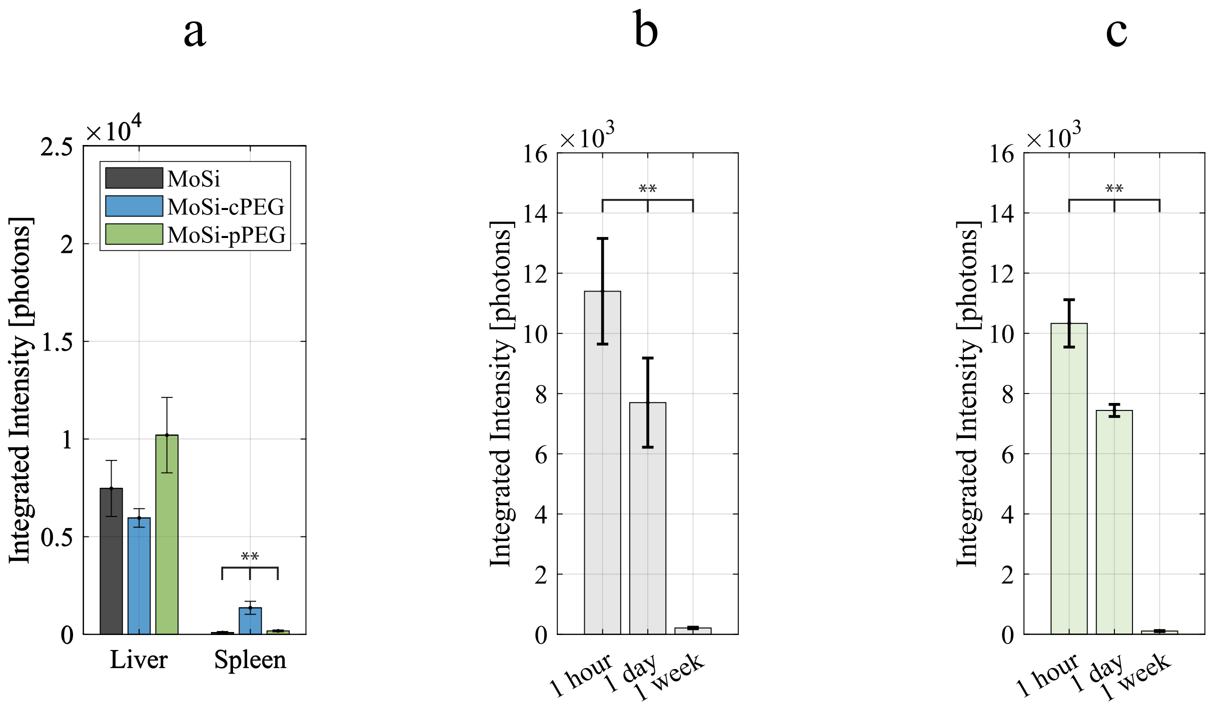

Fig. S8.

***In Vivo* Quantitative Studies**. (**a**) Integrated intensity (XRF photons) of 2D-segmented liver and spleen of mice injected with MoSi (in grey), MoSi-cPEG (in blue), and MoSi-pPEG (in green) NPs (n = 3, ± SD), imaged 24 h after nanoparticle injection. Integrated overall intensity (XRF photons) of mice injected with (**b**) MoSi (in grey) and (**c**) MoSi-pPEG (in green) NPs (n = 3:3:2, ± SD), acquired after 1 h, 1 day, and 1 week after nanoparticle injection. Significant difference (ANOVA analysis) between the groups was indicated when **P < 0.005.

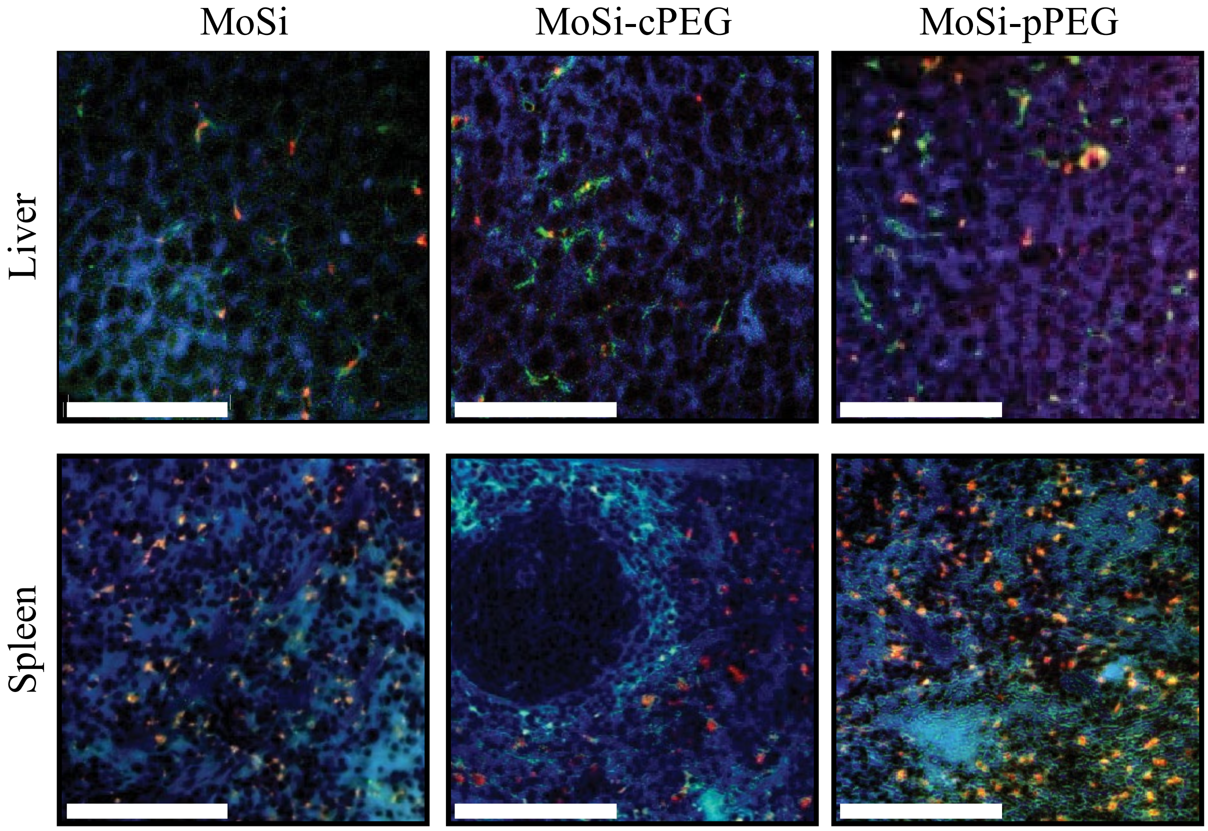

Fig. S9.

Immunofluorescence Staining at the 1-Week Timepoint. Confocal images of liver (top) and spleen (bottom) tissues of mice intravenously administered with MoSi (left column), MoSi-cPEG (middle column), or MoSi-pPEG (right column) euthanized 1 week after nanoparticle (NP) administration. Immunofluorescence staining (Actin/Phalloidin-Alexa Fluor 405 in blue, F4/80- Alexa Fluor 488 in green, Cy5.5 from NPs in red). Scale bars, 100 µm.
